# Supplementary material for: A Comprehensive Assessment of Ultraviolet-Radiation-Induced Mutations in Flammulina filiformis Using Whole-Genome Resequencing
Source: J Fungi (Basel). 2024 Mar 20;10(3):228. doi: 10.3390/jof10030228 (PMC10971301; doi:10.3390/jof10030228)
Supplement: Supplementary file 1 [file jof-10-00228-s001.zip › Supplementary Material S8/KEGG annotation/out/64381550635650.os/KO/out_map/map04724.html]

KEGG PATHWAY: Glutamatergic synapse - Reference pathway


|  |  |
| --- | --- |
| **Glutamatergic synapse - Reference pathway** |  |

[
Pathway menu
| Organism menu
| Pathway entry
| Show description
| User data mapping
]

Glutamate is the major excitatory neurotransmitter in the mammalian central nervous system(CNS). Glutamate is packaged into synaptic vesicles in the presynaptic terminal. Once released into the synaptic cleft, glutamate acts on postsynaptic ionotropic glutamate receptors (iGluRs) to mediate fast excitatory synaptic transmission. Glutamate can also act on metabotropic glutamate receptors (mGluRs) and exert a variety of modulatory effects through their coupling to G proteins and the subsequent recruitment of second messenger systems. Presynaptically localized Group II and Group III mGluRs are thought to represent the classical inhibitory autoreceptor mechanism that suppresses excess glutamate release. After its action on these receptors, glutamate can be removed from the synaptic cleft by EAATs located either on the presynaptic terminal, neighboring glial cells, or the postsynaptic neuron. In glia, glutamate is converted to glutamine, which is then transported back to the presynaptic terminal and converted back to glutamate.

|  |  |
| --- | --- |
| Reference pathway | 100% |
